# Assessment of IL28 (rs12980275) and (rs8099917) Frequency in Recurrent Ocular Herpes Simplex Virus (HSV) Infection

**DOI:** 10.3390/life15030389

**Published:** 2025-03-01

**Authors:** Borivoje Savic, Bozidar Savic, Tanja Kalezic, Bojana Dacic-Krnjaja, Veljko Milosevic, Sanja Petrovic Pajic, Vesna Maric, Tiana Petrovic, Svetlana Stanojlovic

**Affiliations:** 1 University Clinical Centre of Serbia, University Eye Hospital, Pasterova 2, 11000 Belgrade, Serbia; 2 Institute of Veterinary Medicine of Serbia, Janisa Janulisa 14, 11000 Belgrade, Serbia; 3 Faculty of Medicine, University of Belgrade, 11000 Belgrade, Serbia; 4 Institute of Forensic Medicine “Milovan Milovanović”, University of Belgrade-School of Medicine, 31a Deligradska Str., 11000 Belgrade, Serbia

**Keywords:** genotype IL28, rs12980275, rs8099917, keratitis, herpes simplex virus type 1 (HSV-1)

## Abstract

(1) Introduction: The main way of spreading the herpes simplex virus 1 (HSV-1) is through direct contact, as the virus enters the host via mucous membranes. Ocular infection can occur as a primary infection or as a recurrent one. The movement of HSV-1 along the ophthalmic branch of the fifth cranial nerve from its latency phase in the trigeminal ganglion and its activation represent a process influenced by various symbiotic factors, such as environmental conditions and the host’s genetic characteristics. The aim of this study was to assess the frequency of IL28 (rs12980275) and (rs8099917) in recurrent ocular HSV infections. (2) Materials and methods: The study included 60 patients aged over 18, of both sexes, all of whom had a history of herpes simplex labialis (HSL). Patients were tested for HSV-1-specific IgG antibodies, and seropositive individuals were genotyped for single nucleotide polymorphisms (SNPs) rs12980275 and rs8099917. A total of 57 seropositive patients were included in the study. (3) Results: A statistically significant association was found between recurrent HSV keratitis (HSK) and heterozygous GT rs8099917 and homozygous TT rs8099917, as well as heterozygous AG rs12980275 and homozygous AA rs12980275 (*p* < 0.01). Interestingly, patients with homozygous GG polymorphism for both genotypes GG rs8099917 and GG rs12980275 did not develop recurrent HSV keratitis. (4) Conclusion: The most frequent SNP variations in patients with recurrent HSV disease were heterozygous AG rs12980275 (61.40%) and heterozygous GT rs8099917 (52.63%). Patients with recurrent HSV keratitis lacked the homozygous GG polymorphism in both GG rs8099917 and GG rs12980275 genotypes, suggesting that HSV-seropositive individuals expressing these genotypes may have lower predisposition to develop recurrent stromal HSV keratitis.

## 1. Introduction

Herpetic keratitis is predominantly a recurrent disease caused by the reactivation of the HSV-1 virus from latency. Interleukins play a significant role in the fight against viral infections by catalyzing immune responses and assisting in the elimination of viruses from the body. Interleukin-28 (IL28) is one such interleukin that contributes to the body’s antiviral defense mechanisms [1]. IL28 has two isoforms, IL28A (IFN-λ2) and IL28B (IFN-λ3), and is part of the type III interferon family of interleukins. Certainly, the presence of type 1 interleukin (IL-1) plays the most significant role in controlling HSV-1 viral replication [2]. Antiviral activity associated with lambda interferons (IFN-λ) and the activation of interferon-stimulated genes (ISG) has been demonstrated [3]. This form of action represents the fundamental mechanism for the inhibitory effect of IFN-λ on the HSV-1 virus. The genomic arrangement of IFN-λ genes (IL28B, IL28A, and IL29) and the location of key single nucleotide polymorphisms (SNPs) on chromosome 19, within a close region of the IL28A and IL28B genes, indicate a possible association between the polymorphisms (rs12980275 and rs8099917) and herpes keratitis.

Virus replication during reactivation depends on the coding of several important classes of viral genes [4]. These genes encode three groups of proteins: very early (IE); early (E); and late (L). All of these are important and act in cascade. IE proteins and E proteins together with DNA replication are required to activate the L gene. HSV-1 certainly uses immune evasion mechanisms, and the individual’s immune responses are activated to control viral replication and keep it latent. IFN-lambda (λ) is extremely important for maintaining latency and reducing HSV-1 virus replication [5,6,7,8,9].

Several surface glycoproteins (gB, gD, gH and gL) are very important for virus penetration. They are necessary for the connection of the cell membrane and together they form a complex that makes this possible. All four glycoproteins play a very important role in the relationship between HSV infection and the host’s immune system. These glycoproteins are responsible for all host immune responses in response to HSV infection. They are a key mechanism for understanding the relationship between the host immune system and HSV infection.

## 2. Materials and Methods

In this experimental study, written informed consent was obtained from all participants. The research adhered to the guidelines established by the ethics committee and followed the principles set out in the Helsinki Declaration. This in vitro experimental study involved human blood samples. All experimental procedures were carried out in compliance with the regulations of the Institutional Review Board, ensuring ethical standards were maintained.

Sixty participants, aged over 18 years and of both sexes, took part in the study. All participants completed a questionnaire indicating a history of recurrent herpes simplex labialis (HSL) infections. Blood samples were collected from all patients to confirm HSV-1 IgG positivity. Fifty-seven participants were seropositive for HSV-1 and included in the study.

Twenty-six of these patients also had a history of recurrent HSV keratitis, which resulted in severe corneal scarring and a decline in visual acuity. All patients were followed for at least one year, from January to December 2023, at the Eye Clinic of the University Clinical Center of Serbia, Belgrade. Genotyping for IL28 (rs12980275) and (rs8099917) was performed in a laboratory that holds a TQM quality system at the Institute for Veterinary Medicine, Belgrade.

The clinical examination of patients included the following:

- Medical history and physical examination;
- Immunological analyses;
- Molecular genotyping techniques.

### 2.1. Inclusion and Exclusion Criteria

The study involved participants with a history of recurrent HSL infections and confirmed HSV-1 IgG seropositivity. Individuals who tested negative for anti-HSV-1 IgG antibodies were excluded from the study, leaving a primary sample of 57 participants.

Recurrences of herpetic keratitis with stromal form and consequent corneal scarring due to stromal appearance of herpetic keratitis were included.

Additionally, the study included patients with ocular disease associated with herpetic keratitis, corneal scarring, and neovascularization, resulting in significant visual impairment (less than 6/60 on the Snellen scale).

All patients with a history of associated ocular diseases, previous eye surgeries, as well as systemic diseases, neurological disorders, and autoimmune diseases were excluded.

### 2.2. Immunological Analyses

An ELISA test was performed from a serum sample for all patients to detect seropositivity for HSV-1 IgG antibodies. No special preparation was required for obtaining the 5 mL serum samples. Commercial isozyme tests (GenWay Biotech, Inc., San Diego, CA, USA) were used to detect the type of specific IgG antibodies to specific glycoproteins. The significance threshold was set according to the specifications of the commercial IgG antibody test.

### 2.3. Genotype Determination

Five milliliters of peripheral venous blood was collected from each participant for genotyping of IL28 (rs12980275) and (rs8099917). Total DNA was isolated using the commercial KIAamp DNA Blood Mini Kit (QIAGEN, Hilden, Germany), following the manufacturer’s instructions. The single nucleotide polymorphisms (SNPs) for IL28 (rs12980275) and (rs8099917) located on chromosome 19, were analyzed through genotyping.

**Figure 1:**
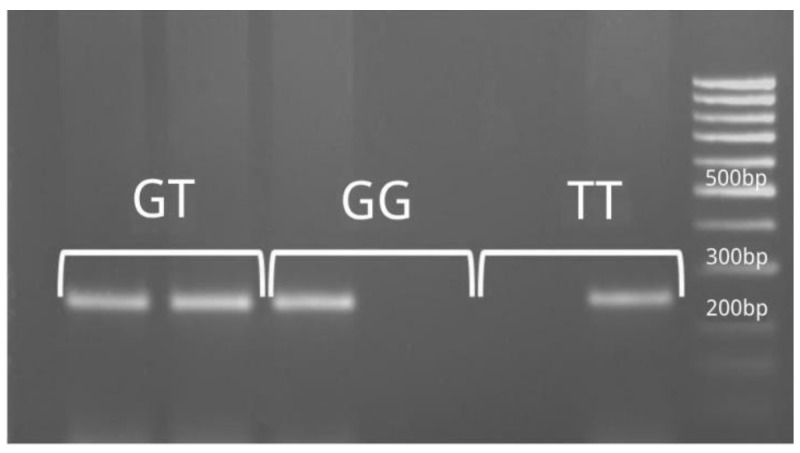
Polymorphism rs8099917—determination of genotypes (PCR product size 249 bp).

**Figure 2:**
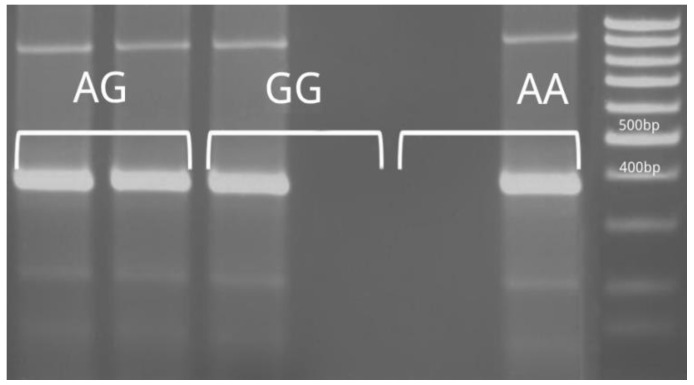
Polymorphism rs12980275—determination of genotypes (size of PCR product 393 bp).

Specific primers were used that allowed us to amplify the region within the corresponding polymorphism using the specified primers (Table 1) [10]. Visualization of PCR products was conducted on a 2% agarose gel. (Figure 1, Figure 2) The presence of the requested sequences served as the basis for genotyping the requested polymorphisms. The amplification procedure was carried out with an initial denaturation step at 95 °C for 5 min, followed by 35 cycles at 95 °C for 30 s, 58 °C for 45 s, 72 °C for 60 s and a final extension at 72 °C for 10 min.

For statistical data analysis, the SPSS^®^ (IBM Corp. Released 2011. IBM SPSS Statistics for Windows, Version 20.0. Armonk, NY, USA: IBM Corp.) software was used. The threshold for statistical significance was established at *p* < 0.01. The Chi-square test of independence was used to evaluate the potential link between rs12980275 and rs8099917 genotypes and the occurrence of HSK infection, with *p*-values less than 0.01 deemed statistically significant. Descriptive statistical methods were used to present the results. By calculating the distribution of genotypes, it was assessed whether the investigated gene variation is in Hardy–Weinberg equilibrium (HWE) with a probability of *p* > 0.05.

The total sample was based on the results of studies by other authors who investigated the influence of the examined genotype. The χ^2^ test was used to determine the difference in the frequency and distribution of different genotypes in the test groups. Each examined polymorphism was individually correlated in order to determine which genotype has the most significant role in predicting HSK. All statistical analyses were tested for a probability greater than *p* < 0.01 with a study power of 0.08 and a number of 57 patients as the corresponding power of a study.

## 3. Results

### 3.1. Clinical Characteristics

A total of 57 participants were enrolled in the study aged 18 years and older, representing both sexes. To homogenize the study group, all participants, regardless of having a history of recurrent herpetic keratitis, had experienced repeated episodes of labial herpes, as confirmed by a questionnaire they voluntarily completed. Furthermore, all participants were tested for IgG anti-HSV-1 antibodies. Fifty-seven out of sixty participants tested positive for HSV-1 IgG and were included in the study. Based on the analysis of medical records and clinical examination, it was determined that twenty-six patients had a history of recurrent herpetic keratitis with significant scarring and corneal vascularization due to stromal herpetic keratitis. These corneal changes led to a considerable reduction in visual acuity in these patients. The remaining thirty-one participants had only a history of recurrent labial herpes without ocular involvement.

### 3.2. Distribution of IL28 Genotype Polymorphisms (rs12980275 and rs8099917)

Host genotypes IL28 (rs12980275) and (rs8099917) were evaluated in all participants. For the IL28 (rs12980275) genotype, the highest prevalence was found in the heterozygous AG rs12980275 (61.40%), followed by homozygous GG rs12980275 (24.56%) and homozygous AA rs12980275 (14.04%).

For the IL28 (rs8099917) genotype, the highest prevalence was observed in the heterozygous GT rs8099917 (52.63%), followed by homozygous TT rs8099917 (12.28%) and homozygous GG rs8099917 (35.09%).

The genotypes GT rs8099917 and TT rs8099917 were significantly associated with the occurrence of recurrent herpetic stromal keratitis (HSK). The GT rs8099917 genotype was present in 30 patients, of whom 21 exhibited recurrent HSK. The heterozygous TT rs8099917 genotype was found in 7 patients, of whom 5 displayed recurrent HSK. Interestingly, the homozygous GG rs8099917 genotype was present in 20 patients, who did not exhibit recurrent HSK, although all these patients experienced multiple episodes of labial herpes (HSL). Additionally, the genotypes AG rs12980275 and AA rs12980275 were significantly associated with the occurrence of recurrent HSK. The heterozygous AG rs12980275 genotype was found in 35 patients, of whom 21 showed recurrent HSK. The heterozygous AA rs12980275 genotype was present in 8 patients, of whom 6 displayed recurrent HSK. Notably, the homozygous GG rs12980275 genotype was found in 14 patients, none of whom showed recurrent HSK.

## 4. Discussion

Herpes simplex virus type 1 (HSV-1) is the most common viral pathogen in the global population. The number of infected carriers continues to rise, reaching as high as 60–90% of individuals over the age of 50 [11]. In most cases, this infection is asymptomatic. After the primary infection, the virus remains in the body for life, remaining latent in the nearby nerve ganglia. Herpetic keratitis occurs in various forms, primarily depending on the depth of the virus’s invasion into the corneal tissue. The majority of herpetic keratitis cases represent recurrent disease, which results from the reactivation of HSV-1 from latency in the nearby nerve ganglia [12].

In our study, we analyzed the IL28 (rs12980275) and (rs8099917) polymorphisms in patients seropositive for HSV-1 who have a history of recurrent HSV-related diseases, including HSV keratitis and/or labial herpes. The most frequent variations of SNPs were the heterozygous AG rs12980275 and heterozygous GT rs8099917 genotypes. Patients with recurrent HSV keratitis did not exhibit the homozygous GG polymorphism in both GG rs8099917 and GGr s12980275 genotypes. These results suggest that it is extremely important to examine the genotype to potentially predict the further development of the disease. Patients with constant reactivation of labial herpes with homozygous genotypes are clearly not prone to develop GG rs8099917 and GG rs12980275 recurrent herpes keratitis. These genotypes probably establish latent control of the virus, but not in terms of quantitative replication and reactivation, but in terms of where reactivation will occur. This is a significant finding because recurrent herpes keratitis (HSK) is associated with possible severe corneal damage. Certainly, the influence of other immunological-genetic factors, as well as epigenetic influences, should not be overlooked. The epigenetic status of HSV-1 virus reactivation has been thoroughly described primarily at the level of the active latency-associated transcript (LAT) region, which is directed at chromatin arrangement without coding for known proteins.

The GG rs8099917 and GG rs12980275 host genotypes probably develop a symbiotic effect with the HSV-1 virus primarily at the level of the manifestation form. All this suggests that the clinical manifestation and the site of reactivation from the trigeminal ganglia can be associated with different genotypes, including other factors that influence the genotype of the host, such as the microenvironment, the host’s epigenome, the macroenvironment, virus virulence and the influence of the external environment. It is certainly necessary to further investigate this host’s genotype, especially what leads to possible reactions in terms of gene transcription stimulated by interferon (ISG). Perhaps the ISG levels in patients with such genotypes are responsible for the site and method of HSV-1 virus reactivation. Host genes can certainly influence the outcome and severity of HSV-1 infection influencing innate resistance factors as well as immune system function and its response to various pathogens. Our study aims to raise questions about the immune susceptibility to HSV-1 infection, which is modulated or dependent on polymorphisms in genes that control the effector functions of cytotoxic T-cells and NK cells (natural killer cells).

By linking several studies that monitored the genetic characteristics of the host, it is concluded that genetic polymorphisms can negatively affect toll-like receptor 3 (TLR3)—UNC-93B, which is responsible for the presentation of type I and type III IFNs [13,14,15]. This is very important in the development of immunity and the progression of HSV-1 infection, which can be life-threatening.

Several studies have explored the connection between host gene polymorphisms and the severity of HSV recurrence [16,17,18,19]. An Italian cohort study found that the TT genotype was correlated with more severe HSV-1 herpes labialis [16]. Moreover, lower serum levels of IFN-λ were associated with the increased severity of herpes labialis. The genotype of the host is important for the coding of IFN-λ, which is predominantly expressed in type 2 myeloid dendritic cells. Their role is particularly important as the first line of defense against viral infections within the epithelium itself. IFN-λ levels are directly related to several neurological diseases.

Certain polymorphisms in these patients increased IFN-λ levels, altering the immune response to HSV-1 infection. Manipulation of the host’s immune system provides the reactivated virus with partial relief from the host’s immune surveillance. Evolution has evidently balanced this possibility in such a way that the host immune system still controls viral replication, maintaining the balance. In our opinion, this likely represents a shift in that balance. The polymorphism in our study likely shifts this balance in a similar manner, altering the immune response to infection. Additionally, host polymorphisms correlate with HSV seropositivity and serum IFN-λ levels in healthy individuals and patients with Alzheimer’s disease (AD). AD patients carrying the T allele displayed higher serum IFN-λ concentrations compared to healthy controls, while those with the C/C genotype had the highest levels of anti-HSV-1 serum antibodies. This further supports the notion that the IFN-λ3 polymorphism influences the immune response to HSV-1 [20].

In order for the HSV-1 virus to achieve lifelong infection, it requires an immune evasion strategy. The virus itself has developed various methods to manipulate the host’s immune system. One typical example is based on molecular mimicry. Most viruses encode homologs of cellular interleukins (IL), chemokines, or chemokine receptors. The virus mimics the host within the host’s cells—by integrating its DNA into the host’s genome, it preserves the host cell’s “resources”, preventing exhaustion by other pathogens that might otherwise compromise the cell’s survival. The host genotype or immune response to the infection reflects symbiotic relationship between the virus and the host. In our study, this symbiosis in GG rs8099917 and GG rs12980275 genotypes maintains the direction of viral replication. Based on our findings, we conclude that the location and form in which the virus will appear depends largely on the symbiosis between host genetics and the virus itself. This symbiosis is a dominant factor in reactivation process and the place of reactivation in addition to the genetic features of the virus itself. When discussing the virulence of the virus, it has distinct features that are challenging to quantify. Due to the unpredictable nature of the virus, it remains unclear which path it will take for its clinical manifestation. This highlights the importance of studies like ours, which expand our understanding of the host genotype and its ability to symbiotically interact with the infection, aiming to cause the least possible damage. Where and in what form the virus will appear depends more on the nature of the host. When it comes to the virulence of the virus itself, the virus has its own characteristics, which are very difficult to quantify. Given the unpredictable nature of the virus, it is unclear which pathway it will choose for its clinical manifestation. During the latency phase, the virus tries not to disrupt normal physiological processes in the cell. The essence of the mechanism of reactivation and virus latency is the result of the virus’s epigenetic nature, its genetics, the host’s genetics, and the influence of external factors. All of this together forms a complex activation and reactivation mechanism, which is certainly associated with other factors that have yet to be discovered in modern virology. We linked the genetic traits of the host—specifically the genotype—to the onset of the disease. These results suggest that it is extremely important to examine the genotype to potentially predict the further development of the disease. Patients with constant reactivation of labial herpes with homozygous genotypes are clearly not prone to develop GG rs8099917 and GG rs12980275 recurrent herpes keratitis. These genotypes probably establish latent control of the virus, but not in terms of quantitative replication and reactivation, but in terms of where reactivation will occur. This is a significant finding because recurrent herpes keratitis (HSK) is associated with possible severe corneal damage. Certainly, the influence of other immunological-genetic factors, as well as epigenetic influences, should not be overlooked.

The epigenetic status of HSV-1 virus reactivation has been thoroughly described primarily at the level of the active latency-associated transcript (LAT) region, which is directed at chromatin arrangement without coding for known proteins. Type III interferons have an effect on HSV-1 infection and the response to this infection by various cells contributes to local defense against HSV-1 as a pathogen. The replication and production mechanism of different interferons during active HSV-1 infection is very similar. However, differences are noted: first of all, that the replication of type III interferon relies more on the NF-κB signaling pathway compared to type I interferon. Our results primarily serve as a prediction for future genetic studies that will also consider new polymorphisms. The limitations of the study are reflected in the sample size and the genetic characteristics of the causative agent itself. Further research should also examine the genomic status of the virus causing the clinical presentation of the infection. Future studies should monitor the depth of viral penetration in patients with herpetic keratitis, paying particular attention to the severity of the clinical presentation and more clearly linking it to various polymorphisms.

## 5. Conclusions

The most common SNP variations observed in patients with recurrent HSV disease are heterozygous AG rs12980275 and heterozygous GT rs8099917. Our study found that the homozygous GG rs12980275 and GG rs8099917 genotypes represent SNP variations that are not associated with recurrent herpetic disease (HSK). Our results suggest that HSV-1-seropositive individuals expressing GG homozygotes may not have a tendency to develop recurrent herpetic keratitis (HSK). Polymorphisms in the IL28 genotypes (rs12980275 and rs8099917) appear to play a role in shaping the clinical presentation and severity of recurrent HSV-1 infection.

## Figures and Tables

**Table 1:** Nucleotide sequences of the primers used in this study. SNP: single-nucleotide polymorphism.

IL28	Nucleotide Sequence	Size of the PCR Product.
rs8099917	Gen (sense) 5′-CCCACTTCTGGAACAAATCG-3′ G (antisense) 5′-CATGGTTCCAATTTGGGTGA C-3′ T (antisense) 5′-CATGGTTCCAATTTGGGTGA A-3′	249 bq
rs12980275	Gen (antisense) 5′-ATGATCATAGCTCATTGCAGC-3′ A (sense) 5′-A GAAGTCAAATTCCTAGAAAC A-3′ G (sense) 5′-A GAAGTCAAATTCCTAGAAAC G-3′	393 bq

## Data Availability

The authors will provide the raw data supporting the conclusions of this article upon request.
